# Literary and Journalistic Notes

**Published:** 1905-05

**Authors:** 


### LITERARY AND JOURNALISTIC NOTES.

D. Appleton & Company announce the publication at short intervals of a translation of "Die Deutsche Klinik," which is being brought out in parts in the German language. Professors Leyden and Klemperer are the editors of the German work, and the articles are written by such well-known authorities as Leube, Ewald, Boad, Baginsky, Liebermeister, Eichhorst, Strumpell, Jurgensen, Ehrlich, Grawitz, Gerhardt, Loeffler, Krafft-Ebing, Hoffa, Oertner, Kaposi and many others whose names are familiar.

It is the plan to publish this work in several volumes, to be translated and edited under the general supervision of Dr. Julius L. Salinger, of Philadelphia, Pa. Each volume in the series will have a special editor.

The first volume of "Modern Clinical Medicine"—"Infectious Diseases" will be published on the 3d of May, 1905. This work will be edited with annotations by Dr. J. C. Wilson, professor of medicine at the Jefferson Medical College, Philadelphia. The second volume, which will appear shortly after the first, will consist of "Constitutional Diseases and Diseases of the Blood."

---

THE *Iris*, volume VIII., 1905, published by the Iris Association, University of Buffalo, is a superb specimen of college editing and bookmaking. The illustrations, consisting of half-tones and drawings in black and white, are artistic in design and execution. The text will be appreciated, most undoubtedly, by the members of the several classes to whom the "roasts" particularly apply. The entire volume is a credit to the editors and especially to the editor-in-chief, Mr. Herman D. Andrews, '05.

---

*Medical Notes and Queries*, is the title of a periodical brochure edited by Dr. Henry W. Cattell, of Philadelphia. It contains many useful paragraphs on a variety of subjects of importance to physicians. The publication office is at Lancaster, Pa.

---

THE first annual report of the Henry Phipps Institute for the study, treatment, and prevention of tuberculosis, embraces the period from February 1, 1903, to February 1, 1904. It is a quarto volume of 265 pages and contains much that is of importance, pertaining to the theme of which it treats. It is another indication of progress in staying the inroads of consumption.

---

NOTES for the Guidance of Authors in the Submission of Manuscripts to Publishers is sent out by the Macmillan Company, 64 Fifth avenue, New York. It is a most useful little brochure, and every physician who makes any pretense of writing for the medical press should obtain it, no matter how much or how little his experience as a writer. It contains hints of value on points that are often overlooked even by veteran authors. Price, 25 cents.

---

THE New York State Hospital for the Care of Crippled and Deformed Children, located at Tarrytown, has sent out its report for the year 1904. Bishop Potter is the president of the board of managers and Newton M. Shaffer is the surgeon-in-chief. This hospital is one of the most deserving charities in the state and is worthy the attention of every philanthropic individual. Since the publication of this report the hospital has been removed to West Haverstraw, Rockland county, the new site being situated within convenient distance from the West Shore and Erie railway stations.

